# Thrombelastography Guides Transfusion Strategy

**Published:** 2009-06

**Authors:** Henning Bay Nielsen

**Affiliations:** *Departments of Anesthesia, Gentofte Hospital and Rigshospitalet, University of Copenhagen, Denmark*

**Keywords:** abdominal aortic aneurysm, blood loss, coagulation, TEG

## Abstract

During surgery for an abdominal aortic aneurysm, coagulation may be impaired and the use of thrombelastography (TEG) is described in six patients with a perioperative blood loss of 3L. During surgery blood products were infused but not platelets. When the patients were admitted to the intensive care unit, the TEG report demonstrated a lowered MA value indicating impaired function of platelets. The use of TEG may guide transfusion strategy.

## INTRODUCTION

In response to emergency surgery for a ruptured abdominal aortic aneurysm patients develop thrombocytopenia (1) and a pro-coagulant state may increase morbidity (2). A simple protocol to optimize transfusion strategy increased survival rate in patients with a ruptured abdominal aortic aneurysm (3). Patients undergoing elective surgery for non-ruptured abdominal aortic aneurysm may also benefit from a proactive administration of platelets and fresh-frozen plasma, as platelet function may be impaired (4). Thrombelastography (TEG) provides a dynamic and global assessment of the coagulation process (5) but none have reported the use of TEG in patients undergoing elective surgery for abdominal aortic aneurysm.

In six patients (five males and one female, age 64 ± 3 yr and weight 73 ± 3 kg; mean ± SEM) TEG was obtained immediately after admission to the intensive care unit. Surgery lasted for 3.2 ± 0.5 hr and anesthesia was sevoflurane and fentanyl administered according to clinical effect. All patients were tracheally intubated and mechanically ventilated to achieve normocapnia according to end-tidal CO_2_. After surgery anesthesia was terminated and when awake patients were extubated and admitted to the intensive care unit for a 24 hr postoperative monitoring. Presented results are evaluated using paired t-test.

During surgery, the total blood loss was 2917 ± 872 ml and the average transfusion strategy included 3 L crystalloid, 1.5 L colloid (macrodex), 4 units fresh frozen plasma and 6 units red blood cells. Infusion of platelets (one unit) was introduced to a patient with a blood loss of 5.7 L. After surgery, the platelet count was decreased to 159 ± 23 × 10^9^/L as compared to 270 ± 29 × 10^9^/L before surgery (*P*<0.05). In the patient with a marked blood loss, TEG R-time was prolonged to 12.3 min (normal range 4 - 8 min) and the patient received additional two units of fresh-frozen plasma. In the other patients, the TEG R-time was 5-7 min and therefore within the normal range. In all patients the platelet function was impaired as the TEG MA variable was lowered to 44 ± 2 mm (normal range 55-73 mm). Thus, within the first hours after admission to the intensive care unit, 1–2 units of platelets were administered to each patient and the platelet count increased to 188 ± 10 × 10^9^/L the day after surgery.

The data suggests, that in response to elective vascular surgery for non-ruptured abdominal aortic aneurysm, coagulation is impaired and platelets should be offered to the patients. The use of TEG may guide postoperative transfusion strategy.
